# The Emerging Role of VEGF-C and the Lymphatic System in Diuretic Resistance

**DOI:** 10.3390/life16101614

**Published:** 2026-09-26

**Authors:** Laura Elena Zamora-Cervantes, Viridiana Galicia Galicia, Diego Sánchez-Hernández, Enzo Vásquez-Jiménez, Consuelo Plata, Katy Sánchez-Pozos

**Affiliations:** 1 Department of Nephrology, Hospital Juárez de México, Mexico City 07760, Mexico; lolezamora@gmail.com (L.E.Z.-C.); viri.galicia.galicia@gmail.com (V.G.G.); sahendiego@gmail.com (D.S.-H.); enzo.vas.ji@gmail.com (E.V.-J.); 2 Department of Nephrology and Mineral Metabolism, Instituto Nacional de Ciencias Médicas y Nutrición Salvador Zubirán, Mexico City 14080, Mexico; consuelo.platar@incmnsz.com; 3 Research Division, Hospital Juárez de México, Mexico City 07760, Mexico

**Keywords:** interstitial edema, sodium homeostasis, intrarenal pressure, congestion

## Abstract

Diuretic resistance (DR) amounts to a significant clinical hurdle in patients with heart failure (HF) and cardiorenal syndrome (CRS), as it is linked to persistent congestion and adverse clinical outcomes. Although impaired drug delivery, neurohormonal activation, renal hemodynamic alterations, and tubular adaptation contribute to a diminished natriuretic response, these mechanisms may not completely explain congestion, particularly in patients who appear to achieve an adequate natriuretic response. Current decongestive strategies focus mainly on increasing renal sodium and fluid excretion, leaving other mechanisms such as interstitial fluid mobilization relatively unexplored. The lymphatic system plays an important role in maintaining interstitial fluid and sodium homeostasis and adapts both functionally and structurally in response to elevated interstitial fluid load. Vascular endothelial growth factor-C (VEGF-C), acting primarily via its receptor VEGFR-3, may regulate lymphatic adaptation in response to different stimuli. Recent experimental and clinical observations, largely from observational studies, suggest that disturbances in lymphatic adaptation could cause persistent interstitial congestion; however, the extent of its direct involvement in pharmacological DR, and whether changes in circulating VEGF-C are causative, remain unclear. In this narrative review, we analyze DR mechanisms and evaluate current evidence linking lymphatic function and VEGF-C/VEGFR-3 signaling to congestion. We introduce an interstitial–lymphatic background in which ongoing congestion heightens lymphatic demand, while maladaptive responses may hinder interstitial fluid clearance and promote renal interstitial edema. Elevated renal interstitial pressure, in theory, influences renal hemodynamics and sodium handling through a renal tamponade-like effect, potentially causing lowered natriuretic responsiveness. This proposed mechanism remains hypothetical, and further empirical and clinical investigation is warranted. Determining whether VEGF-C acts as a mediator, a compensatory signal, or a biomarker of lymphatic dysfunction could help clarify the role of the interstitial–lymphatic compartment in cardiorenal decongestion and DR.

## 1. Introduction

DR is defined as an attenuated response despite adequate loop diuretic dosing, resulting in decreased sodium and chloride excretion and limiting the ability to achieve euvolemia [1,2]. It is still a challenge in managing HF, CRS, and other edematous disorders, with estimates ranging from 15% to 68% [3,4]; persistent congestion strongly predicts adverse outcomes [5]. Traditionally, DR has been attributed to tubular adaptation, neurohormonal activation, altered pharmacokinetics, and intravascular congestion [6,7,8,9]. Consequently, current decongestive strategies have primarily concentrated on optimizing natriuresis and reducing intravascular congestion [10]. Nevertheless, considerable interindividual variability in diuretic responsiveness persists, and adequate natriuresis does not always translate into complete decongestion, suggesting that additional mechanisms may contribute to persistent congestion.

Recognizing congestion as a multicompartmental phenomenon is essential, in which the interstitium and lymphatic system act as active compartments for sodium homeostasis and can redistribute sodium independently of the vascular space [11,12].

The lymphatic system maintains fluid and sodium homeostasis [13] and adapts to increased interstitial fluid load through a dynamic, time-dependent process involving early functional responses and slower structural remodeling [14,15]. When increased lymphatic demand persists, post-developmental lymphangiogenesis may assist in sustaining drainage capacity [16]. This response is regulated predominantly by VEGF-C/VEGFR-3 signaling and modulated by mechanical, inflammatory, and osmotic stimuli that may coexist under pathological conditions, such as increased filling pressure characteristic of congestion [17,18,19]. However, sustained hemodynamic and inflammatory stress may overwhelm these adaptive mechanisms, potentially leading to maladaptive lymphatic remodeling and impaired interstitial fluid clearance. Emerging evidence suggests that impaired lymphatic adaptation may cause persistent interstitial congestion; however, its direct contribution to true pharmacological DR is still unclear [20,21,22]. This review analyzes the interstitial–lymphatic compartment and VEGF-C/VEGFR-3 signaling as a potential framework for understanding persistent congestion and their potential contribution to DR.

## 2. Methods

A structured literature search was conducted in PubMed/MEDLINE and Scopus to identify studies examining the relationship between VEGF-C, cardiac lymphatic biology, congestion, and diuretic response in heart failure. Four complementary searches were performed in each database using combinations of terms associated with VEGF-C, heart failure, congestion, and lymphatic biology: (1) VEGF-C, heart failure; (2) VEGF-C, congestion; (3) lymphatic, heart failure, and congestion; (4) VEGF-C, lymphatic, heart failure. No additional search algorithms, filters, or database-specific search fields were applied. The initial search was limited to human studies to identify available clinical evidence. We performed searches on 21 August 2026. Supplementary Table S1 provides the search terms, search date, and number of records retrieved for each search.

We combined records retrieved from both databases and removed duplicates before screening. Two reviewers independently screened titles and abstracts using the eligibility criteria. Reviewers then assessed potentially relevant reports in full text. We considered only reports with an available full-text publication for inclusion in the primary evidence synthesis. Reviewers resolved disagreements through discussion and consensus. No reports were unavailable for retrieval.

Studies were eligible if they provided original experimental, physiological, observational, or interventional evidence relevant to VEGF-C/VEGFR-3 cardiac or peripheral lymphatic biology, congestion, or diuretic/decongestive response in heart failure. We selected studies based on their relevance to the review question, study design, methodological characteristics, and contribution to mechanistic or clinical evidence. We excluded reviews, case reports or series, study protocols without outcome data, book chapters, corrigenda, abstracts without a corresponding full-text publication, and studies addressing unrelated diseases or mechanisms from primary evidence synthesis.

Given the nature of this review, which encompassed heterogeneous experimental, mechanistic, observational, and interventional studies, no formal risk-of-bias assessment was applied. Instead, we considered study design, sample size, methodological characteristics, the directness of the evidence, and major study limitations during synthesis as reported in Table 1 and Supplementary Table S1.

We also examined the reference lists of selected articles to identify additional potentially relevant studies. We present the study selection process using a PRISMA 2020 flow diagram (Figure 1).

## 3. Diuretic Resistance and Persistent Congestion

Although established criteria remain lacking, DR is generally defined as an attenuated response despite adequate loop diuretic dosing. Consequently, sodium and chloride excretion fail and impair decongestion, predisposing to persistent or recurrent fluid overload that often requires dose escalation or combination diuretic therapy [2,3,5,6,23]. However, defining an adequate dose depends on the clinical context [24,25,26]. Diuretic response is the kidney’s efficiency in generating urine following its administration [27]. Because intravenous loop diuretics reach peak serum concentration at 2 h and remain active for about 6 h, clinicians can assess the response within the first 2 h after dosing [28]. To define an adequate diuretic response, the American Heart Association recommends achieving a spot urine sodium > 50 mmol/L or a urine output > 150 mL/hour [3]. In contrast, the European Society of Cardiology sets the spot urine sodium threshold at > 70 mmol/L [5].

Diagnosing DR remains challenging because of the lack of a standardized definition and the limitations of established metrics for measuring diuresis and natriuresis in clinical practice. Furthermore, weight-based metrics for evaluating persistent congestion capture only changes in total body water [29], which is often inappropriately used as a surrogate for volume overload. This concept is not synonymous with congestion, which may result from inadequate natriuresis, impaired fluid redistribution and tissue fluid mobilization, or a combination of these mechanisms [30]. Consequently, the precise prevalence of DR remains unclear. Some studies estimate that 15–30% of patients with HF exhibit DR, with variation driven by comorbidities such as impaired renal function [3], reaching an incidence as high as 68% in CRS [4]. Although detailed data on the chronic kidney disease (CKD) population remains limited [31,32], DR itself is associated with poor clinical outcomes and persistent congestion [32,33].

### Established Pathophysiological Mechanisms

DR is multifactorial, comprising both extratubular and intratubular mechanisms [2,6]. Extratubular factors involve pharmacokinetic alterations, which may reduce the amount of active drug reaching the tubular lumen [34,35]. Intestinal edema may impair oral absorption, whereas extracellular fluid expansion and ascites in CRS or liver disease can increase the volume of distribution. Hypoalbuminemia further alters the distribution and renal delivery due to high protein binding [8]. Reduced renal perfusion and impaired tubular secretion—as in CKD—may additionally limit drug delivery by competing with organic anion transporters [36]. Collectively, these mechanisms can decrease the intratubular concentration available to inhibit the sodium–potassium–chloride cotransporter (NKCC2)—the mechanism of action of loop diuretics—despite apparently adequate dosing [37].

Even after achieving adequate intratubular concentrations, further mechanisms may still blunt the natriuretic response. Intratubular mechanisms account for over 70% of this response variability [38], driven by neurohormonal activation and the renin–angiotensin–aldosterone system (RAAS). In HF and CRS, systemic and renal inflammation may amplify neurohormonal activity through increased sympathetic nervous system activity and RAAS stimulation. Pro-inflammatory cytokines and adipokines—leptin, tumor necrosis factor-alpha (TNF-α), interleukin-6 (IL-6) and adiponectin—induce inflammation and apoptosis, followed by fibrosis, consequently promoting endothelial dysfunction and altering renal microvascular and tubular function [39,40]. RAAS activation subsequently enhances proximal tubular sodium reabsorption following diuretic exposure [38].

Furthermore, chronic exposure to loop diuretics induces distal nephron remodeling characterized by hypertrophy and hyperplasia of epithelial cells, leading to upregulation of the sodium–chloride cotransporter (NCC) and the epithelial sodium channel (ENaC) [6,38,41]. DR may be further aggravated by loop diuretic-induced hypochloremia, which triggers the with-no-lysine kinase (WNK) pathway and enhances sodium reabsorption via NCC [7,8,42]. Additionally, the depletion of luminal chloride—a vicious cycle driven by loop diuretics—promotes sodium reabsorption in the collecting duct via pendrin in conjunction with the sodium-dependent chloride/bicarbonate exchanger (NDCBE) [6,8,43], thereby perpetuating diuretic resistance.

Accordingly, the traditional framework of DR focused on tubular sodium handling and intravascular congestion; however, congestion is not synonymous with fluid overload, and increasing evidence supports the concept that it is a multicompartmental process involving both the interstitium and the lymphatic circulation [11,20].

## 4. Congestion as a Multicompartmental Phenomenon

Congestion results from active interactions between the intravascular and extravascular compartments [30]. In HF and CRS, sodium retention and volume redistribution from the splanchnic reservoir to the central circulation, caused by increased cardiac and systemic venous filling pressures, contribute to intravascular congestion [30,44,45]. In other conditions, such as liver disease, reduced plasma oncotic pressure may further promote fluid movement into the interstitial compartment [46].

Current decongestive strategies primarily target renal sodium excretion and intravascular volume reduction. However, natriuresis, diuresis, and interstitial decongestion are not necessarily equivalent responses. Some patients remain clinically congested despite an apparently effective natriuretic response [5,25,26]. Moreover, aggressive diuresis may reduce intravascular volume alongside hypotension without fully resolving peripheral edema [47], suggesting that fluid redistribution into the interstitial compartment could contribute to decongestion failure.

The interstitium may not just be a passive fluid compartment but a functional regulator of sodium and fluid homeostasis [20]. Sustained elevation in venous pressure increases capillary hydrostatic pressure and promotes fluid filtration into the interstitium [22]. Dysfunction of the endothelial glycocalyx and glycosaminoglycan (GAG) network induced by inflammation and oxidative stress may further alter tissue sodium and fluid distribution [9,48] with additional sodium release from GAG-binding sites, increasing capillary filtration and leakage and thereby increasing the functional demand on lymphatic drainage [13,49].

The lymphatic system provides a route for returning excess interstitial fluid to circulation. Increased interstitial fluid formation initially stimulates lymphatic flow and adaptive reactions [14]; however, when lymphatic transport capacity is overwhelmed, fluid accumulates within the interstitial compartment [50]. In HF and CRS, elevated central venous pressure may also reduce the pressure gradient for lymphatic return, additionally impairing tissue fluid clearance [34]. Because the interstitial compartment has a higher fluid retention capacity than the intravascular space [51,52], incomplete mobilization of this fluid may cause persistent congestion despite reductions in intravascular volume.

This distinction has clinical implications. Persistent intravascular and interstitial congestion after hospitalization for acute heart failure (AHF) independently predicts rehospitalization and mortality (HR 1.69, 95% CI 1.15–2.49, and 1.25–3.41, respectively) [53]. In addition, interstitial phenotype may be particularly difficult to resolve [54]. Nonetheless, persistent congestion after diuretic therapy should not be interpreted as true DR, as an adequate renal natriuretic response may coexist with inefficient mobilization and clearance of interstitial fluid [52]. This dissociation supports considering the lymphatic system as a potential determinant of interstitial decongestion, although its direct contribution to impaired renal diuretic responsiveness remains to be established.

## 5. Kidney Lymphatics and Lymphangiogenesis

The lymphatic system maintains fluid homeostasis by returning proteins, electrolytes, and approximately 8 L of fluid per day from the interstitium to the venous circulation [14,55]. Lymphatic transport is initiated by the lymphatic endothelial cells (LECs), which feature a glycocalyx-coated luminal surface and specialized, discontinuous “button-like” junctions that allow the exchange of fluid and solutes [56]. Because of fluid flow, they are constantly exposed to shear stress [57]; anchoring filaments sense these changes in interstitial pressure and direct flow unidirectionally through the lymphangions toward the lymphatic ducts [56,58,59,60].

Kidney lymphatic vessels are distributed mainly in the cortical interstitium adjacent to renal vasculature, where they help regulate fluid balance by reabsorbing electrolytes, proteins, and inflammatory mediators [61].

Under physiological conditions, kidney lymphatics help maintain renal interstitial pressure and fluid homeostasis [51,61,62,63].

### 5.1. Time-Dependent Dynamics of Lymphatic Adaptation and Lymphangiogenesis

Lymphatic adaptation to increased interstitial fluid load is an active and time-dependent process involving both early functional responses and slower structural remodeling. Increases in interstitial pressure can initially enhance lymph formation and transport by mechanically opening initial lymphatic junctions and regulating vessel contractility via nitric oxide (NO) and prostaglandins (PG) [51,64,65,66,67]. These responses may increase lymphatic transport without forming new vessels. In contrast, maintaining LECs integrity and function through post-developmental lymphangiogenesis requires LEC activation, proliferation, and remodeling and therefore evolves over a longer time scale [15,16,21,68,69].

Experimental cardiovascular models suggest that lymphatic adaptation follows a dynamic and context-specific temporal course. In pressure-overload models, cardiac lymphatic remodeling develops over weeks and varies with hemodynamic stress [20,22]. Notably, different mouse strains showed distinct temporal patterns, ranging from early lymphatic expansion at 2–3 weeks to pronounced lymphangiogenesis after 6–8 weeks [21,22]. Conversely, although lymphatic drainage function initially increased, it was impaired by week 8, driven by VEGF-C/VEGFR-3 signaling [20].

These results indicate that structural lymphangiogenesis does not follow a fixed temporal sequence; rather, it depends on the magnitude and duration of tissue stress, as well as VEGF-C/VEGFR-3 signaling. Functional adaptation of pre-existing lymphatics may precede structural lymphangiogenesis, while sustained stress can lead to maladaptive lymphatic remodeling. Whether renal lymphatics follow a similar temporal pattern during acute and chronic congestion remains unknown.

Structural adaptation requires promoting signaling lymphangiogenesis when lymphatic demand remains high. VEGF-C/VEGFR-3 route represents a major regulator [70]. Mechanical, osmotic, and inflammatory stimuli may converge on this pathway, complementing signals that maintain lymphatic flow [71].

### 5.2. Mechanisms Regulating Lymphangiogenesis

Post-developmental lymphangiogenesis is regulated through integrating mechanical, inflammatory, and osmotic signals arising from the interstitial microenvironment. Although these stimuli activate partially distinct pathways, VEGF-C/VEGFR-3 signaling represents a central regulatory axis promoting LECs’ proliferation, migration, survival, and vessel remodeling. Thus, rather than acting as an isolated pathway, VEGF-C/VEGFR-3 may integrate changes in interstitial fluid flow, inflammation, and sodium accumulation into an adaptive lymphatic response.

Mechanical stimuli are particularly important during increased interstitial fluid formation. Increased interstitial flow and laminar shear stress can activate VEGFR-3 phosphorylation in LECs, and high levels of this receptor subsequently sensitize endothelial cells to shear forces [72,73]. The calcium influx mediated by Piezo-type mechanosensitive ion channel component 1 (PIEZO1) contributes to the regulation of transcription factor Prospero homeobox 1 (PROX1) and genes involved in lymphatic valves maintenance [15,57,74]. At the same time, the junctional mechanosensory complex comprising platelet endothelial cell adhesion molecule (PECAM-1) and vascular endothelial cadherin activates the downstream phosphatidylinositol 3-kinase (PI3K)/Akt pathway. Integrins (α1β1, αVβ3, α9, α5β1) provide an additional mechanism through signaling extracellular matrix stretching of LECs [52,57].

Inflammatory stimuli provide a second regulatory pathway. In experimental kidney injury, pro-inflammatory cytokines—including TNF-α, transforming growth factor-β1 (TGF-β1), and interferon-gamma (IFN-γ)—infiltrate macrophages and proximal tubular epithelial cells [75,76,77]. These cytokines can modulate VEGF-C signaling and promote lymphangiogenesis [78].

Finally, interstitial sodium accumulation may provide an osmotic stimulus for lymphangiogenesis. Researchers have suggested that hypertonic conditions may activate nuclear factor of activated T cells 5 (NFAT5) signaling, which has been associated with increased VEGF-C expression [79,80,81]. Together, these pathways provide a biological framework through which changes in the interstitial environment may regulate lymphatic adaptation.

Although it remains unclear whether lymphangiogenesis is beneficial, experimental evidence indicates that newly formed lymphatic vessels commonly exhibit irregular architecture [54,82]. This may compromise vessel integrity, leading to interstitial leakage, impaired drainage, and persistent interstitial edema [83]. However, vessel structure appears to depend on the microenvironment [13,14,84]. In contexts of abnormally high shear stress, PROX1 expression decreases, leading to a loss of LECs’ identity [85]. Because PROX1 is required for VEGFR-3 expression, VEGFR-3 levels are subsequently reduced, leading to an impaired LEC response to shear stress and reduced functionality [57,86].

Therefore, failure of these adaptive functions during chronic congestion provides a biological rationale to consider lymphatic dysfunction an emerging contributor to DR, as discussed in the following sections.

## 6. Evidence Linking Lymphatics and VEGF-C During Congestion

Evidence linking lymphatic dysfunction and VEGF-C/VEGFR-3 signaling to congestion derives mostly from experimental models and a limited number of human studies. Importantly, evidence of impaired lymphatic drainage during congestion or the role of VEGF-C/VEGFR-3 signaling does not necessarily establish a causal relationship with diuretic resistance (Table 2).

### 6.1. Experimental Evidence of VEGF-C/VEGFR-3 Signaling During Congestion

Experimental evidence provides the strongest mechanistic evidence supporting a role of VEGF-C/VEGFR-3 signaling in lymphatic adaptation. In models of pressure-overload, VEGF-C up-regulation promotes compensatory lymphangiogenesis. It limits myocardial edema and inflammation [20], whereas VEGFR-3 inhibition attenuates lymphangiogenesis and worsens tissue edema and inflammatory infiltration, activating cardiac remodeling and fibrosis [21]. However, these responses appear to be context- and time-dependent, and experimental evidence also indicates that lymphatic dysfunction may occur despite preserved VEGF-C concentrations, suggesting that impaired downstream VEGFR-3 signaling or LECs dysfunction may contribute independently [87]. Collectively, these data derive primarily from cardiac experimental models and cannot be directly extrapolated to renal lymphatics or human DR.

### 6.2. Human Evidence of Lymphatic Dysfunction and Congestion

Human studies suggest that lymphatic abnormalities may accompany congestion, although they do not establish VEGF-C/VEGFR-3 as the underlying mechanism. Indocyanine green lymphography has identified altered lower-extremity lymphatic flow and drainage patterns in patients with HF [89]. More recently, lower-extremity lymphatic flow was associated with diuretic response in AHF [90]. Importantly, these findings show an association between peripheral lymphatic function and decongestive response, but do not show that impaired lymphatic drainage causes DR or that VEGF-C mediates the observed abnormalities.

The potential relevance of the interstitial–lymphatic compartment during congestion may be further supported by emerging interventional strategies targeting interstitial decongestion. The DELTA-HF trial showed the feasibility and short-term safety of the eLym system—a catheter-based therapy to decompress the venous angle—for improving congestion and diuretic response in patients with AHF [91]. Although these findings support the interstitial compartment as a possible therapeutic target, they should not be interpreted as evidence that lymphatic modulation improves diuretic responsiveness.

### 6.3. Human Evidence of VEGF-C/VEGFR-3 and Congestion

Human evidence evaluating VEGF-C remains limited and predominantly observational. In AHF, lower circulating VEGF-C concentrations have been associated with higher peripheral edema (30% versus 13%, *p*= 0.02) and ascites (22% versus 9%; *p* = 0.006), whereas higher concentrations were associated with lower mortality (HR 0.8, 95% CI 0.49–0.94; *p* = 0.019) [92]. In parallel, VEGF-C concentrations have been associated with hypervolemic CKD patients and correlated with bioimpedance [93]. These observations suggest that circulating VEGF-C may reflect alterations in lymphatic, renal, or systemic homeostasis; however, they do not establish whether reduced VEGF-C is a cause, consequence, or biomarker of congestion.

Collectively, current evidence supports the biological plausibility of an association between lymphatic dysfunction, VEGF-C/VEGFR-3 signaling, and congestive states. Still, several important gaps prevent establishing a causal route toward DR. First, most mechanistic evidence derives from experimental cardiac rather than renal models. Second, human lymphatic studies have primarily evaluated peripheral lymphatic function and cannot establish whether similar abnormalities occur within the renal lymphatic network. Third, available human VEGF-C studies are observational and do not determine whether altered circulating concentrations represent a causal mediator. Finally, persistent interstitial congestion and impaired pharmacological diuretic responsiveness are connected but distinct phenomena, and current evidence does not show that lymphatic dysfunction directly reduces tubular responsiveness to loop diuretics. Therefore, we proposed an interstitial–lymphatic framework.

## 7. An Interstitial-Lymphatic Framework in Cardiorenal Congestion

Current models of DR focus on neurohormonal activation, impaired tubular sodium handling, intravascular volume expansion, and elevated venous pressures [3]. While these mechanisms explain most cases of diuretic resistance, they do not account for persistent tissue edema in a substantial proportion of patients despite apparently adequate natriuresis [88].

Impaired lymphatic adaptation of the renal lymphatic network may first cause persistent interstitial congestion; subsequently, increased renal interstitial pressure could alter renal hemodynamics and tubular sodium handling, thereby contributing to DR. However, this remains largely hypothetical and requires direct experimental and prospective clinical validation (Figure 2).

### 7.1. Chronic Congestion and Lymphatic Demand

Sustained venous congestion and sodium accumulation may increase capillary filtration and renal interstitial hydrostatic pressure, increasing the demand for lymphatic drainage.

### 7.2. Progressive Lymphatic Dysfunction

Persistently increased lymph flow, together with chronic inflammation, abnormal mechanical stress, and altered VEGF-C/VEGFR-3 signaling, may promote maladaptive lymphatic remodeling. Experimental studies suggest these processes may lead to LEC dysfunction and reduced drainage, together with possible alterations in lymphatic contractility induced by loop diuretics [50,94].

### 7.3. Renal Interstitial Edema

If lymphatic adaptation becomes insufficient, progressive interstitial edema could increase renal interstitial hydrostatic pressure, creating a “renal tamponade”—a proposed concept in which fluid accumulation within the relatively non-compliant renal capsule may compress the renal parenchyma, possibly impairing renal hemodynamics, glomerular filtration rate (GFR), and sodium excretion [95]. Its contribution to true DR, however, remains hypothetical.

### 7.4. Functional Consequences

Impaired lymphatic adaptation could cause persistent congestion despite continued natriuresis. Sustained congestion may, in turn, further increase venous and interstitial pressures, creating a perpetuating cycle in which lymphatic dysfunction and congestion reinforce one another. Whether this proposed feed-forward mechanism occurs in humans remains to be established.

## 8. Discussion

The central premise emerging from this review is not that lymphatic dysfunction is an established mechanism of DR, but that the conventional DR paradigm may incompletely capture the process that determines effective decongestion. Renal natriuretic response and resolution of congestion represent connected yet separate physiological processes. Although inadequate sodium excretion is central to true pharmacological DR, adequate natriuresis does not necessarily ensure effective mobilization and clearance of fluid from the interstitial compartment. This distinction provides the conceptual basis for considering the interstitial–lymphatic compartment in patients with persistent congestion.

Current evidence supports different levels of inference within this system. Experimental studies support the role of VEGF-C/VEGFR-3 signaling in lymphangiogenesis and lymphatic adaptation, showing that mechanical, inflammatory, and osmotic stimuli modulate lymphatic remodeling. Human studies provide complementary but substantially more limited evidence. At the same time, abnormalities in peripheral lymphatic flow have been associated with congestion and diuretic response, while circulating VEGF-C concentrations have been associated with edema, fluid overload, and clinical outcomes. However, these observations neither establish that peripheral lymphatic disturbances reflect renal lymphatic function nor demonstrate that VEGF-C dysregulation causes impaired pharmacological responsiveness to loop diuretics.

The biological interpretation of VEGF-C within this structure also requires caution. Reduced circulating VEGF-C should not necessarily be interpreted as evidence of impaired lymphangiogenesis, nor should increased concentrations be assumed to indicate an effective adaptive response. VEGF-C may function as a mediator of lymphatic remodeling, a compensatory signal reflecting increased lymphatic demand, or a biomarker of underlying systemic or renal alterations. Moreover, lymphatic dysfunction may occur despite preserved VEGF-C concentrations if downstream VEGFR-3 signaling, LECs’ mechanotransduction, vessel architecture, or contractile function is impaired. Longitudinal assessment of VEGF-C together with direct measures of lymphatic function will therefore be necessary to establish its mechanistic and clinical significance.

From a clinical perspective, the immediate value of this system lies in phenotyping of decongestive failure. Distinguishing inadequate natriuresis from persistent interstitial congestion despite adequate sodium excretion may identify biologically distinct phenotypes that require different therapeutic approaches. Emerging strategies to improve interstitial fluid mobilization or lymphatic drainage provide proof of concept that this compartment may be therapeutically accessible; however, they should not be interpreted as evidence supporting VEGF-C manipulation or as proof that lymphatic dysfunction causes DR. More future studies should therefore integrate serial measurements of urinary sodium and diuretic efficiency with objective assessment of intravascular and interstitial congestion, renal function, and lymphatic transport. Particularly relevant will be studies evaluating the temporal relationship between congestion, VEGF-C/VEGFR-3 signaling, and lymphatic adaptation, as well as measuring renal lymphatic flow and renal interstitial pressure. Such approaches will be necessary to determine whether lymphatic dysfunction is a cause, consequence, or marker of persistent congestion and whether it can secondarily influence true diuretic responsiveness.

## 9. Conclusions

In conclusion, DR and persistent congestion are closely related but should not be considered interchangeable phenomena. Recognized mechanisms of true DR primarily encompass impaired drug delivery, neurohormonal activation, altered renal hemodynamics, and distal tubular sodium adaptation. However, effective natriuresis does not invariably translate into complete decongestion, underscoring a major gap in our current understanding of the pathways governing interstitial fluid mobilization and clearance.

At present, evidence linking the lymphatic system and VEGF-C/VEGFR-3 signaling to DR remains scarce and largely indirect. Most evidence comes from animal models, whereas human studies are limited, observational, and largely restricted to peripheral rather than renal lymphatic function. Consequently, we cannot yet establish whether lymphatic dysfunction or dysregulated VEGF-C/VEGFR-3 signaling directly drives pharmacological DR. Rather than proving a causal link, these limitations underscore an unaddressed mechanistic gap and support exploring the interstitial–lymphatic compartment as a hypothesis-generating framework.

Within the present framework, we hypothesize that maladaptive lymphatic responses contribute primarily to persistent interstitial congestion. In contrast, any downstream effects on renal hemodynamics, tubular sodium handling, or secondary diuretic unresponsiveness remain speculative. Studies are essential to test and refute this hypothesis. Until more evidence emerges, VEGF-C/VEGFR-3 signaling and lymphatic dysfunction continue as subjects for mechanistic investigation rather than validated determinants or therapeutic targets in DR.

## Figures and Tables

**Figure 1:**
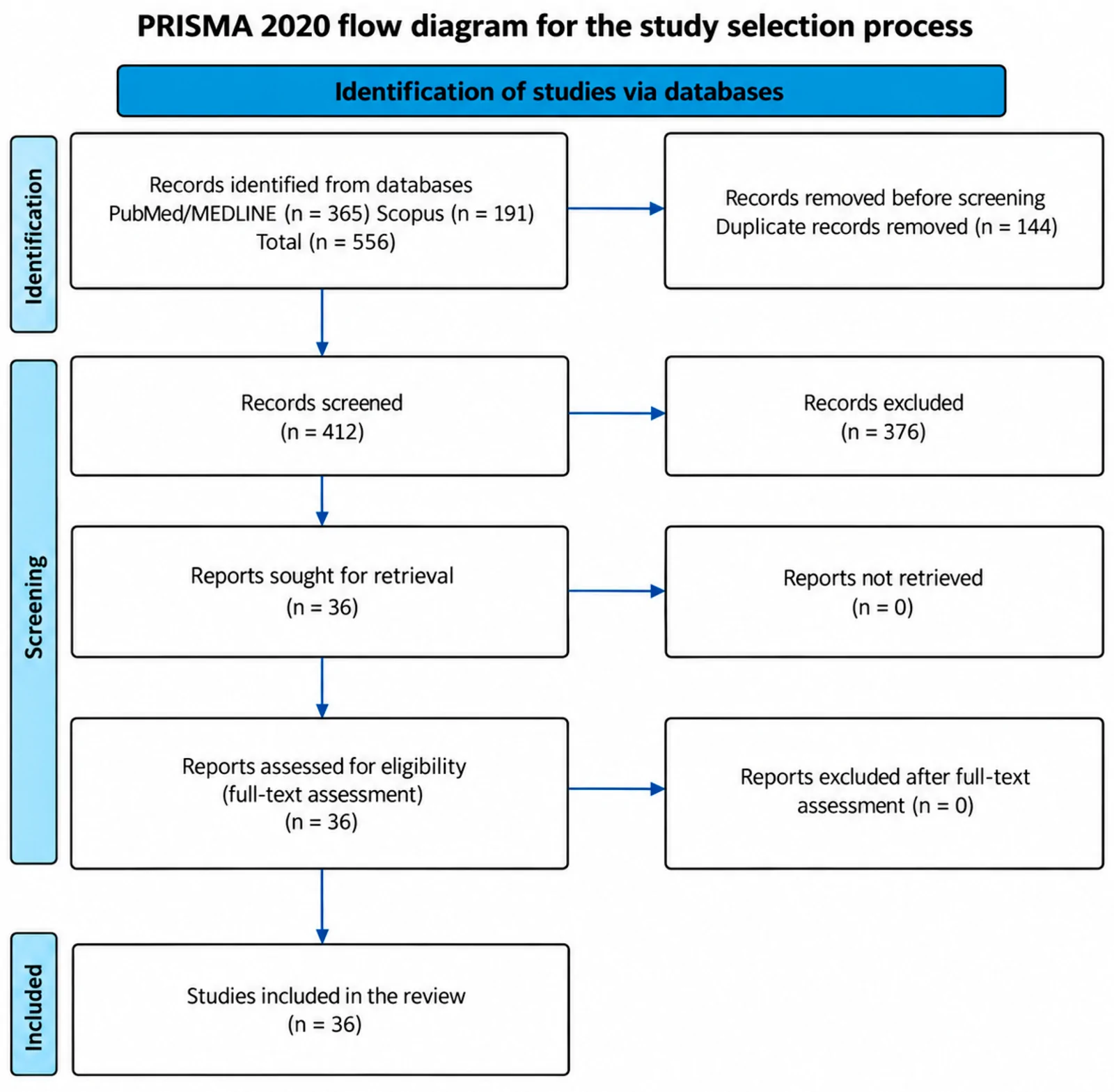
PRISMA 2020 flow diagram for the study selection process. Preferred Reporting Items for Systematic Reviews and Meta-Analyses (PRISMA) flow diagram of the study selection process. Searches were limited to PubMed/MEDLINE and Scopus and were conducted on 21 August 2026. After removing duplicate records, 36 full-text reports met the eligibility criteria and were included in the review. Screening exclusions include records excluded during the title/abstract and detailed relevance assessment.

**Figure 2:**
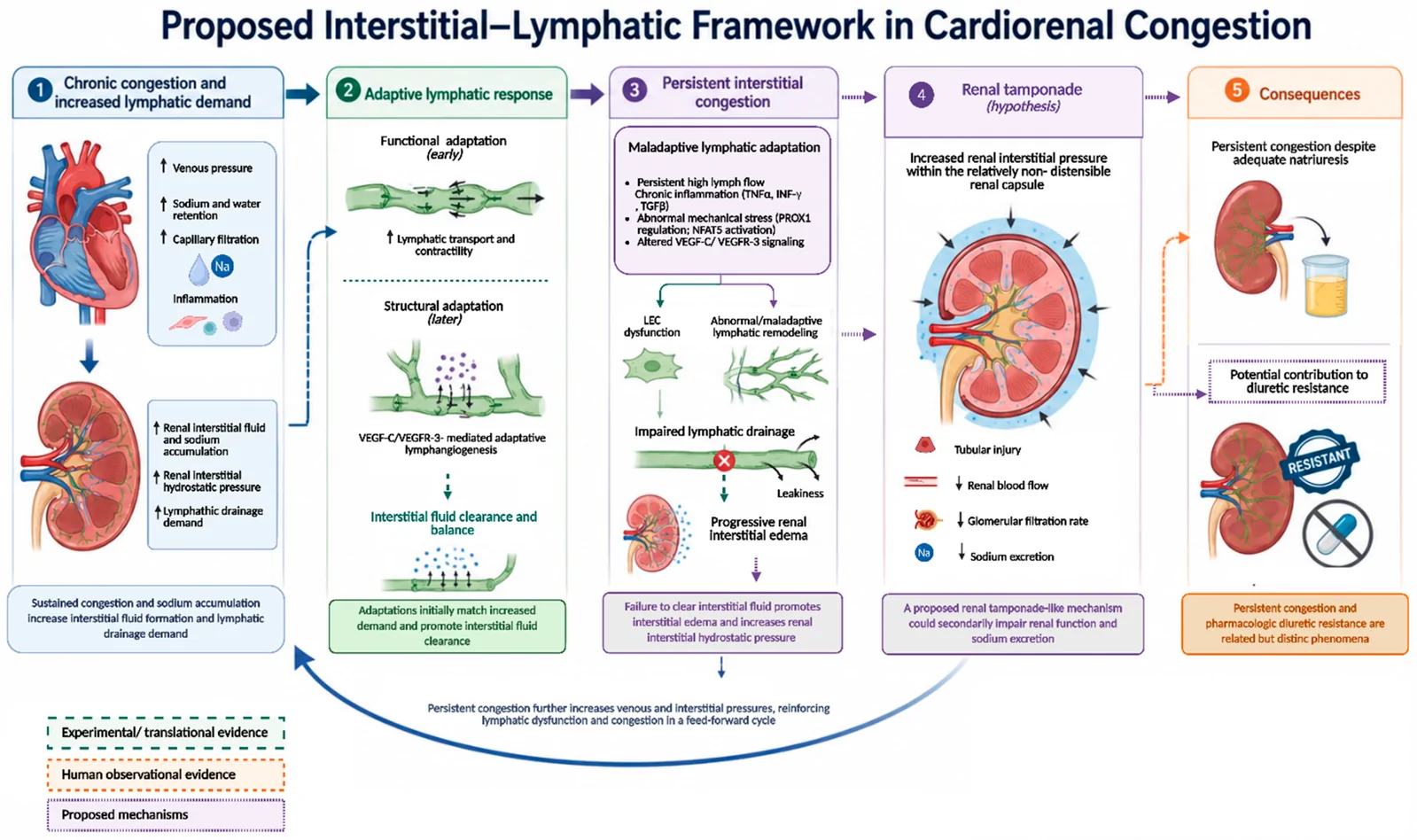
Proposed Interstitial–Lymphatic Framework in Cardiorenal Congestion. Color coding: green, experimental/translational evidence; orange, human observational evidence; purple, proposed mechanisms. Abbreviations: Na+, Sodium; VEGF-C, Vascular Endothelial Growth Factor-C; VEGFR-3, Vascular Endothelial Growth Factor Receptor 3; TNF-α, Tumor Necrosis Factor-Alpha; INF-γ, Interferon-Gamma; TGF-β, Transforming Growth Factor-β1; PROX1, Prospero Homeobox 1; NFAT5, Nuclear Factor of Activated T cells 5; LEC, Lymphatic Endothelial Cells. Created in BioRender. zamora, L. E. (2026): https://BioRender.com/sc59ff9.

**Table 1:** Search strategies and retrieved records.

Database	Search	Search Terms	Search Date	Records Retrieved
PubMed/MEDLINE	Search 1	“VEGF-C”, “heart failure”	21 August 2026	42
PubMed/MEDLINE	Search 2	“VEGF-C”, “congestion”	21 August 2026	8
PubMed/MEDLINE	Search 3	“Lymphatic”, “heart failure”, “congestion”	21 August 2026	285
PubMed/MEDLINE	Search 4	“VEGF-C”, “lymphatic”, “heart failure”	21 August 2026	30
PubMed/MEDLINE	Total	--	21 August 2026	365
Scopus	Search 1	“VEGF-C”, “heart failure”	21 August 2026	45
Scopus	Search 2	“VEGF-C”, “congestion”	21 August 2026	7
Scopus	Search 3	“Lymphatic”, “heart failure”, “congestion”	21 August 2026	115
Scopus	Search 4	“VEGF-C”, “lymphatic”, “heart failure”	21 August 2026	24
Scopus	Total	--	21 August 2026	191
TOTAL	-	--	21 August 2026	556

Abbreviations: VEGF-C, Vascular Endothelial Growth Factor C.

**Table 2:** Evidence supporting the role of VEGF-C/VEGFR-3 and lymphatic dysfunction in congestion and diuretic response.

Study	Population/Model	Main Findings	Evidence Provided	Main Limitations
Experimental VEGF-C/VEGFR-3 evidence				
Lin et al., 2021 [20]	Pressure-overload HF in mice; cardiac lymphatic density/remodeling; using LYVE-1, VEGFR-3 and PDPN	In mice, VEGF-C/VEGFR-3 signaling was increased during early hypertrophy but reduced in failing hearts. VEGF-C156S administration enhanced cardiac lymphangiogenesis, reduced cardiac edema and attenuated hypertrophy, fibrosis, inflammation and dysfunction. In humans, serum VEGF-C concentrations were lower in patients with HF than normal controls.	Provides mechanistic experimental evidence that VEGF-C/VEGFR-3 signaling promotes adaptive cardiac lymphangiogenesis and protects against pressure-overload HF, together with preliminary human evidence of lower circulating VEGF-C in HF.	Animal study; no assessment of diuretic response or renal lymphatic function. The human component was small and observational and did not assess lymphatic function, congestion severity or diuretic response.
Su et al., 2025 [21]	Pressure-overload HF in mice Cardiac and peripheral lymphatic remodeling and transport	VEGFR-3 inhibition impaired adaptive lymphangiogenesis and worsened cardiac inflammation/remodeling, whereas VEGF-C enhanced lymphangiogenesis, peripheral lymphatic drainage and cardiac function.	Additional mechanistic evidence that VEGF-C/VEGFR-3 signaling can modulate lymphatic adaptation and HF phenotype.	Animal study; therapeutic implications remained preclinical.
Guo et al., 2025 [87]	HFpEF in mice and human myocardial tissue Cardiac lymphatic structure/function using LYVE-1/VEGFR-3; expression of LYVE-1, PROX1, PDPN; functional drainage and immune cells	HFpEF was associated with early structural and functional cardiac lymphatic impairment, despite stable VEGF-C levels.	Lymphatic dysfunction may occur independently of reduced VEGF-C availability, through impaired VEGFR-3 trafficking/signaling and LECs metabolic dysfunction.	Animal study, human component based on myocardial tissue and therefore provides structural/molecular evidence.
Human lymphatic and congestion data				
Rossitto et al., 2020 [88]	16 HFpEF patients and 16 controls Tissue VEGF-C and VEGFR-3 expression by skin biopsy; lymphatic density and lymphatic drainage by venous-occlusion plethysmography	HFpEF patients had fewer but larger lymphatic vessels, reduced LYVE-1/PROX-1 expression and impaired lymphatic drainage. VEGF-C showed a trend toward lower expression (*p* = 0.06), whereas VEGFR-3 did not differ.	Direct human evidence of structural and functional lymphatic impairment, with suggestive alteration of VEGF-C signaling.	Small sample; peripheral rather than renal lymphatics; VEGF-C difference did not reach statistical significance; no diuretic-response assessment.
Ponikowska et al., 2024 [89]	20 AHF with lower-limb edema, 7 chronic HF, 10 controls Indocyanine-green fluorescent lymphography	Significant lower-extremity lymphatic flow was observed predominantly in patients with AHF and edema; the study established a minimally invasive method for real-time assessment of lymphatic flow	Human evidence that peripheral lymphatic flow can be directly assessed during HF-associated congestion.	Small, single-center methodological study; no VEGF-C or diuretic response assessment
Ponikowska et al., 2025 [90]	65 AHF patients receiving protocolized IV loop diuretics Indocyanine-green lymphography	Greater/faster lower-extremity lymphatic flow was associated with better diuretic response, assessed by cumulative diuresis and weight loss.	Direct human evidence linking lymphatic function with diuretic response.	Small, observational, single-center; VEGF-C cannot be implicated as the mediator.
Nuche et al., 2026 [91]	40 AHF patients with persistent congestion and high loop-diuretic requirements Therapeutic enhancement of thoracic-duct lymphatic drainage using the eLym™ System	Lymphatic drainage therapy was associated with substantial weight loss and improvement in clinical congestion, with relatively stable serum creatinine.	Preliminary interventional human evidence that directly enhancing lymphatic drainage may facilitate tissue decongestion.	Multicenter, single-arm feasibility study; no control group and no VEGF-C assessment.
Human VEGF-C data linking VEGF-C with congestion and clinical outcomes				
Iwanek et al., 2023 [92]	237 patients hospitalized with AHF Serum VEGF-C	Patients in the lowest VEGF-C tertile had more severe lower-extremity edema (30% vs. 13% vs. 20%) and ascites (22% vs. 9% vs. 6%) and higher 1-year mortality.	Strongest available human observational evidence directly linking low circulating VEGF-C with clinical congestion.	Observational; no direct lymphatic-flow measurement and no assessment of diuretic response; causality cannot be established.

Abbreviations: HF, Heart Failure; VEGF-C, Vascular Endothelial Growth Factor-C; VEGF-C156S, engineering variant of VEGF-C with the potential to promote lymphangiogenesis; LYVE-1, Lymphatic Vessel Endothelial Hyaluronan Receptor 1; VEGFR-3, Vascular Endothelial Growth Factor Receptor-3; PROX1, Prospero Homeobox 1; PDPN, Podoplanin; HFpEF, Heart Failure with Preserved Ejection Fraction; LECs, Lymphatic Endothelial Cells; AHF, Acute Heart Failure; IV, Intravenous; VEGF-D, Vascular Endothelial Growth Factor-D; eLym System, eLym™ System (WhiteSwell, Ltd., Galway, Ireland); HR, hazard ratio; SD, standard deviation.

## Data Availability

This study did not create or analyze new data. This article is a narrative review based on previously published, publicly available literature cited throughout the manuscript.

## Supplementary Materials

The following supporting information can be downloaded at: https://www.mdpi.com/article/10.3390/life16101614/s1, Table S1: Additional studies not included in the primary Table 2.

## Abbreviations

The following abbreviations are used in this manuscript:

AHF	Acute Heart Failure
CKD	Chronic Kidney Disease
CRS	Cardiorenal Syndrome
CI	Confidence Interval
DR	Diuretic Resistance
ENaC	Epithelial Sodium Channel
GAG	Glycosaminoglycan
GFR	Glomerular Filtration Rate
HF	Heart Failure
HFpEF	Heart Failure with Preserved Ejection Fraction
HR	Hazard Ratio
IL-6	Interleukin-6
IFN-γ	Interferon-Gamma
LECs	Lymphatic Endothelial Cells
Na+	Sodium
NCC	Sodium–Chloride Cotransporter
NKCC2	Sodium–Potassium–Chloride Cotransporter
NDCBE	Sodium-Dependent Chloride/Bicarbonate Exchanger
NFAT5	Nuclear Factor of Activated T cells
NO	Nitric Oxide
PG	Prostaglandins
PROX1	Prospero Homeobox 1
PECAM-1	Platelet Endothelial Cell Adhesion Molecule
PI3K	Phosphatidylinositol 3-kinase
RIHP	Renal Interstitial Hydrostatic Pressure
RAAS	Renin–Angiotensin–Aldosterone System
TonEBP	Tonicity-responsive Enhancer-binding Protein
TNF-α	Tumor Necrosis Factor-Alpha
TGF- β1	Transforming Growth Factor-β1
VEGF-C	Vascular Endothelial Growth Factor-C
VEGF-D	Vascular Endothelial Growth Factor-D
VEGFR-3	Vascular Endothelial Growth Factor Receptor-3
WNK	With-No-Lysine Kinase
