# Supplementary material for: The Emerging Role of VEGF-C and the Lymphatic System in Diuretic Resistance
Source: Life (Basel). 2026 Sep 26;16(10):1614. doi: 10.3390/life16101614 (PMC13705914; doi:10.3390/life16101614)
Supplement: Supplementary file 1 [file life-16-01614-s001.zip › life-4504201-supplementary.pdf]

Supplementary Table S1. Additional studies not included in the primary Table 1.

All studies marked as "Include" in the supplied screening file are presented here if they are not represented in the current primary Table 1. Studies retain the evidence-domain classification used for Table 1.

A. Experimental VEGF-C/VEGFR-3 (n = 19)

Study	Population/model	Lymphatic assessment	VEGF-C/VEGFR-3 assessment	Main findings	Contribution to review	Main limitations
Wu et al., 2024	Background: Sepsis-induced cardiomyopathy (SICM), is defined as a global but reversible dysfunction of both the left and right sides of the heart, which plays a significant role in the pathogenesis of sepsis.	lymphatic, lymphangiogenesis, lymphatic vessels, VEGFR3	To evaluate the effects of VEGF-C on LPS-induced SICM, the mice were treated with VEGFC-156S (0.1 mg/kg) via tail vein injection 6 h after LPS challenged and sacrificed 6 h after being treated with VEGFC. To evaluate the effects of the VEGF-C-VEGFR-3 signaling pathway in SICM.	Results: We found that cardiac function was impaired in SICM, along with a significant reduction in the area of lymphatic vessels. Then, we revealed that stimulation of cardiac lymphangiogenesis with vascular endothelial growth factor C (VEGFC) effectively improved cardiac function and promoted neutrophil clearance. Conclusions: Our data show that effective lymphatic vessels are necessary for cardiac function and inflammation resolution in SICM.	Additional experimental evidence supporting VEGF-C/VEGFR-3-related lymphatic remodeling in cardiac disease.	No additional limitation recorded in the screening file.
Luo et al., 2026	HFpEF induced by a 2-hit model (with a high-fat diet [HFD]+L-NAME) in wild-type mice.	lymphatic, lymphangiogenesis, lymphatic vessels, VEGFR3	VCAM1-positive cardiac fibroblasts (VCFs) augmented lymphatic vessel formation and progression in the heart and the lymphangiogenic effect of VCFs were partly mediated by VEGF-C–VEGFR3 signaling, based on cellular experiments using lymphatic endothelial cells.	Methods and Results: We observed the kinetics of cardiac lymphatic vessels in HFpEF induced by a 2-hit model (with a high-fat diet [HFD]+L-NAME) in wild-type mice. Conclusions: Cardiac lymphatic vessels play an important role in remodeling and function in HFpEF progression.	Additional experimental evidence supporting VEGF-C/VEGFR-3-related lymphatic remodeling in cardiac disease.	No additional limitation recorded in the screening file.
Vieira et al. 2018	The mammalian adult heart, however, the persistent influx of immune cells, coupled with the lack of an inherent regenerative capacity, results in cardiac fibrosis.	lymphatic, lymphangiogenesis, LYVE-1, mediastinal lymph	Here, we reveal that stimulation of cardiac lymphangiogenesis with VEGF-C improves clearance of the acute inflammatory response after MI by trafficking immune cells to draining mediastinal lymph nodes (MLNs) in a process dependent on lymphatic vessel endothelial hyaluronan receptor 1 (LYVE-1).	In the mammalian adult heart, however, the persistent influx of immune cells, coupled with the lack of an inherent regenerative capacity, results in cardiac fibrosis. Deletion of Lyve1 in mice, preventing docking and transit of leukocytes through the lymphatic endothelium, results in exacerbation of chronic inflammation and long-term deterioration of cardiac function.	Additional experimental evidence supporting VEGF-C/VEGFR-3-related lymphatic remodeling in cardiac disease.	No additional limitation recorded in the screening file.
Ge et al., 2020	Heart disease patients.	lymphatic, lymphatic vessels, LYVE-1, VEGFR3	The protein expression levels of lymphatic-specific markers, such as vascular endothelial growth factor C (VEGF-C), VEGF receptor 3 (VEGFR3), and lymphatic vessel endothelial hyaluronan receptor 1 (LYVE-1) were detected in mouse heart	The cardiac lymphatic system has been associated with limited research, but it plays an important role in the improvement of myocardial edema and the promotion of fluid balance. The results showed that LCZ696 significantly improved	Additional experimental evidence supporting VEGF-C/VEGFR-3-related lymphatic remodeling in cardiac disease.	No additional limitation recorded in the screening file.

			tissues. LCZ696 also decreased the protein expression levels of VEGF-C, VEGFR3, and LYVE-1 in mouse heart tissues, ameliorated the transport load of lymphatic vessels to macrophages, and improved the remodeling of t	heart dysfunction, cardiac hypertrophy, and fibrosis and inhibited the expression of proinflammatory factors IL-6, IL-1β, and TNF-α in the circulating blood and heart tissues of mice. LCZ696 also decreased the protein expression levels of VEGF-C, VEGFR3, and LYVE-1 in mouse heart tissues, ameliorated the transport load of lymphatic vessels to macrophages, and improved the remodeling of the lymphatic system in the h		
Bai et al., 2022	Mice, resulting in exacerbation of tissue edema, hypertrophy, fibrosis superoxide production, inflammation, and heart failure (HF).	lymphatic, lymphangiogenesis, LYVE-1	In this study, cardiac remodeling models were established by Ang II (1000 ng/kg/min) in VEGFR-3 knockdown (Lyve-1Cre VEGFR-3f/-) and wild-type (VEGFR-3f/f) littermates. Our results indicated that Ang II infusion not only induced cardiac lymphangiogenesis and upregulation of VEGF-C and VEGFR-3 expression in the time-dependent manner but also enhanced proteasome activity, MKP5 and VE-cadherin degradation, p38 MAPK activation, and lymphatic vessel h	Our results indicated that Ang II infusion not only induced cardiac lymphangiogenesis and upregulation of VEGF-C and VEGFR-3 expression in the time-dependent manner but also enhanced proteasome activity, MKP5 and VE-cadherin degradation, p38 MAPK activation, and lymphatic vessel hyperpermeability. Conversely, administration of epoxomicin (a selective proteasome inhibitor) markedly mitigated Ang II-induced cardiac edema, remodeling, and dysfunction; upregulated MKP5 and VE-cadherin expression; inactivated p38 MAPK; and reduced lymphatic vessel hyperpermeability in WT mice, indicating that inhibition of proteasome activity is required to mainta	Additional experimental evidence supporting VEGF-C/VEGFR-3-related lymphatic remodeling in cardiac disease.	No additional limitation recorded in the screening file.
Cui et al., 2023	Background: The dilation of lymphatic vessels plays a critical role in maintaining heart function, while a lack thereof could contribute to heart failure (HF), and subsequently to an acute myocardial infarction (AMI).	lymphatic, lymphangiogenesis, lymphatic vessels, VEGFR3, mediastinal lymph	Macrophages participate in the induction of lymphangiogenesis by secreting vascular endothelial cell growth factor C (VEGF-C), although the precise mechanism remains unclear. Methods: Intramyocardial injections of adeno-associated viruses (AAV9) to inhibit the expression of VEGFR3 (VEGFR3 shRNA) or promote the expression of VEGFR3 (VEGFR3 ORF) in the heart; Myh6-mCherry B6 D2-tg mice and flow cytometry were used to evaluate the number of myocellu	Methods: Intramyocardial injections of adeno-associated viruses (AAV9) to inhibit the expression of VEGFR3 (VEGFR3 shRNA) or promote the expression of VEGFR3 (VEGFR3 ORF) in the heart; Myh6-mCherry B6 D2-tg mice and flow cytometry were used to evaluate the number of myocellular debris in the mediastinal lymph nodes; fluorescence staining and qPCR were used to evaluate fluorescence analysis; seahorse experiment was used to evaluate the level of glycolysis of macrophages; Lyz2Cre,	Additional experimental evidence supporting VEGF-C/VEGFR-3-related lymphatic remodeling in cardiac disease.	No additional limitation recorded in the screening file.

				VEGFCfl/fl, and PFKFB3fl/fl mice were used as a model to knock out the expression of VEGF-C and PFKFB3 in macrophages. Results: The escalation of VEGFR3 in cardiac ti		
Xu et al., 2025	Capsule (NXC) has been applied in the treatment of MI-HF for more than 20 years and has shown clinical effectiveness in improving cardiac function in MI-HF patients.	lymphatic, lymphangiogenesis, lymphatic vessels	The regulatory effects of NXC on the miR-126-3p/SPRED1 and VEGF-C pathways were analyzed by RT-qPCR, Western blotting, and Dual-luciferase assay. Network pharmacology and bioinformatics analyses further revealed that NXC exerted its cardioprotective effects by promoting cardiac lymphangiogenesis through the modulation of the VEGF-C pathway by miR-126-3p/SPRED1.	Results: Our research results demonstrated that NXC notably improved cardiac function in MI-HF mice, facilitated the formation of cardiac lymphatic vessels, reduced the expression of inflammatory factors, and alleviated myocardial fibrosis. Conclusion: The findings of this research indicate that NXC can stimulate the VEGF-C pathway via miR-126-3p/SPRED1 to promote cardiac lymphangiogenesis, thus treating MI-HF. These results offer valuable insights for the development of cardiovascular drugs targeting MI-HF by leveraging the potential of NXC to enhance cardiac lymphangiogenesis.	Additional experimental evidence supporting VEGF-C/VEGFR-3-related lymphatic remodeling in cardiac disease.	No additional limitation recorded in the screening file.
Houssari et al., 2020	Mice, we found that only sustained VEGF (vascular endothelial growth factor)-CC156Stherapy, achieved by adeno-associated viral vectors, increased cardiac lymphangiogenesis, and led to reduced cardiac inflammation and dysfunction by 3 weeks post-MI.	lymphatic, lymphangiogenesis, VEGFR3	Conversely, inhibition of VEGF-C/-D signaling, through adeno-associated viral delivery of soluble VEGFR3 (vascular endothelial growth factor receptor 3), limited infarct lymphangiogenesis. Conclusions: We show that resolution of cardiac inflammation after MI may be accelerated by therapeutic lymphangiogenesis based on adeno-associated viral gene delivery of VEGF-CC156S.	Approach and Results: Comparing adenoviral versus adeno-associated viral gene delivery in mice, we found that only sustained VEGF (vascular endothelial growth factor)-CC156Stherapy, achieved by adeno-associated viral vectors, increased cardiac lymphangiogenesis, and led to reduced cardiac inflammation and dysfunction by 3 weeks post-MI. Conversely, inhibition of VEGF-C/-D signaling, through adeno-associated viral delivery of soluble VEGFR3 (vascular endothelial growth factor receptor 3), limited infarct lymphangiogenesis. Unexpectedly, this treatment improved cardiac function post-MI in both mice and rats, linked to reduced infarct thinning d	Additional experimental evidence supporting VEGF-C/VEGFR-3-related lymphatic remodeling in cardiac disease.	No additional limitation recorded in the screening file.
Luo et al., 2026	SICM pathogenesis and evaluated the therapeutic efficacy of targeted lymphangiogenesis as a	lymphatic, lymphangiogenesis, lymphatic vessels	In vitro assays with lymphatic endothelial cells (LECs) confirmed that this lymphangiogenic	Methods and results: Using a lipopolysaccharide (LPS)-induced SICM mouse model, the	Additional experimental evidence supporting VEGF-C/VEGFR-3-related lymphatic	No additional limitation recorded in the screening file.

	novel strategy to improve poor prognosis.		potency is mediated, at least in part, by vascular endothelial growth factor-C (VEGF-C) secreted by ADRCs.	evolution of cardiac lymphatic kinetics and observed reparative lymphangiogenesis was assessed in SICM. Notably, ADRC-mediated therapeutic lymphangiogenesis restored cardiac function and mitigated adverse pathological remodeling as well as significantly improved the overall prognosis in SICM-affected animals. Conclusion: Our findings highlight the essential role of cardiac lymphatic vessels in improving SICM outcomes and indicate that ADRC-driven therapeutic lymphangiogenesis is a promising and innovative modality for treating this highly severe condition.	remodeling in cardiac disease.	
Chen et al., 2024	Mice with VMC inhibited lymphangiogenesis, while supplementation with VEGF-CC156S depressed it.	lymphatic, lymphangiogenesis, lymphatic vessels, VEGFR3	Macrophage depletion and VEGF-CC156S treatment were used to investigate the connection between macrophages and cardiac lymphangiogenesis. After treatment with AAV-sVEGFR3, lymphangiogenesis was inhibited, leading to worsened cardiac dysfunction and aggravated inflammation.	Adeno-associated virus (AAV) was used to determine the effect of cardiac lymphangiogenesis in VMC. Results: Cardiac inflammation and lymphatic vessel density were both upregulated, peaking on day 7 following CVB3 infection. After treatment with AAV-sVEGFR3, lymphangiogenesis was inhibited, leading to worsened cardiac dysfunction and aggravated inflammation.	Additional experimental evidence supporting VEGF-C/VEGFR-3-related lymphatic remodeling in cardiac disease.	No additional limitation recorded in the screening file.
Su et al., 2025	Mice subjected to transverse aortic constriction.	lymphatic, lymphangiogenesis, VEGFR3	However, the efficiency of systemically delivered VEGFC (vascular endothelial growth factor C) protein inducing cardiac lymphangiogenesis has shown inconsistency across different studies. METHODS: To determine the role of lymphatic remodeling in heart failure, we used adeno-associated virus–mediated gene therapy to either inhibit VEGFR3 (vascular endothelial growth factor receptor 3) expression or enhance VEGFC expression in mice subjected to	METHODS: To determine the role of lymphatic remodeling in heart failure, we used adeno-associated virus–mediated gene therapy to either inhibit VEGFR3 (vascular endothelial growth factor receptor 3) expression or enhance VEGFC expression in mice subjected to transverse aortic constriction. In vitro, studies were further conducted to investigate the role of Yes-associated protein in VEGFC/VEGFR3 signaling in lymphatic endothelial cells. RESULTS: Our study shows that transverse aortic constriction induces dynamic lymphatic remodeling, characterized by an early adaptive cardiac lymphangiogenic	Additional experimental evidence supporting VEGF-C/VEGFR-3-related lymphatic remodeling in cardiac disease.	No additional limitation recorded in the screening file.

				response and enhanced peripheral lymphatic transport		
Xiang et al., 2026	Not reported in screening record.	Not assessed/reported.	Not assessed/reported.	See abstract in screening record.	Additional experimental evidence supporting VEGF-C/VEGFR-3-related lymphatic remodeling in cardiac disease.	Study type/limitations are apparent from the screening record.
Nakanishi et al., 2026	Not reported in screening record.	Not assessed/reported.	Not assessed/reported.	See abstract in screening record.	Additional experimental evidence supporting VEGF-C/VEGFR-3-related lymphatic remodeling in cardiac disease.	Study type/limitations are apparent from the screening record.
Guo et al., 2025	Not reported in screening record.	Not assessed/reported.	Not assessed/reported.	See abstract in screening record.	Additional experimental evidence supporting VEGF-C/VEGFR-3-related lymphatic remodeling in cardiac disease.	Study type/limitations are apparent from the screening record.
Keller et al., 2021	Not reported in screening record.	Not assessed/reported.	Not assessed/reported.	See abstract in screening record.	Additional experimental evidence supporting VEGF-C/VEGFR-3-related lymphatic remodeling in cardiac disease.	Study type/limitations are apparent from the screening record.
Lin et al., 2021	Not reported in screening record.	Not assessed/reported.	Not assessed/reported.	See abstract in screening record.	Additional experimental evidence supporting VEGF-C/VEGFR-3-related lymphatic remodeling in cardiac disease.	Study type/limitations are apparent from the screening record.
Zhang et al., 2019	Not reported in screening record.	Not assessed/reported.	Not assessed/reported.	See abstract in screening record.	Additional experimental evidence supporting VEGF-C/VEGFR-3-related lymphatic remodeling in cardiac disease.	Study type/limitations are apparent from the screening record.
Shimizu et al. 2018	Not reported in screening record.	Not assessed/reported.	Not assessed/reported.	See abstract in screening record.	Additional experimental evidence supporting VEGF-C/VEGFR-3-related lymphatic remodeling in cardiac disease.	Study type/limitations are apparent from the screening record.
Henri et al., 2016	Not reported in screening record.	Not assessed/reported.	Not assessed/reported.	See abstract in screening record.	Additional experimental evidence supporting VEGF-C/VEGFR-3-related lymphatic remodeling in cardiac disease.	Study type/limitations are apparent from the screening record.

B. Lymphatic dysfunction and congestion (n = 8)

Study	Population/model	Lymphatic assessment	VEGF-C/VEGFR-3 assessment	Main findings	Contribution to review	Main limitations
Salah et al., 2025	patients with HF.	lymphatic, thoracic duct	Not assessed/reported.	See abstract in screening record.	Additional evidence linking lymphatic structure/function with cardiac congestion or decongestion.	No additional limitation recorded in the screening file.
Liu et al., 2023	66 patients enrolled, 60 completed the study.	lymphatic	Not assessed/reported.	Methods and results: This was a pre-determined, secondary analysis of the TOLF-HF [The-Optimal-Lymph-Flow for Heart	Additional evidence linking lymphatic structure/function with	No additional limitation recorded in the screening file.

				Failure (TOLF-HF)] study, a two-arm pilot randomized controlled trial evaluating the preliminary effects of the lymphatic exercise intervention in enhancing interstitial decongestion among patients with HF. The study results exhibited that the TOLF-HF intervention were effective in alleviating both physical and psychological symptom distress. The intervention group yielded significantly lower MLHFQ total scores in comparison to the control group.	cardiac congestion or decongestion.	
Kelly et al., 2024	Patients with chronic right-sided heart failure.	lymphatic, lymphatic vessels, thoracic duct	Not assessed/reported.	Results: In total nine patients and nine controls were included. Conclusion: In this small exploratory study, individuals with severe secondary tricuspid valve regurgitation and right-sided heart failure displayed a largely similar lymphatic anatomy and function. Thoracic duct diameter displayed a trend towards increased size in the patient group.	Additional evidence linking lymphatic structure/function with cardiac congestion or decongestion.	No additional limitation recorded in the screening file.
Gharzeddine et al., 2026	Not reported in screening record.	Not assessed/reported.	Not assessed/reported.	See abstract in screening record.	Additional evidence linking lymphatic structure/function with cardiac congestion or decongestion.	Study type/limitations are apparent from the screening record.
Dori et al., 2023	Not reported in screening record.	Not assessed/reported.	Not assessed/reported.	See abstract in screening record.	Additional evidence linking lymphatic structure/function with cardiac congestion or decongestion.	Study type/limitations are apparent from the screening record.
Chabbert et al., 2004	Not reported in screening record.	Not assessed/reported.	Not assessed/reported.	See abstract in screening record.	Additional evidence linking lymphatic structure/function with cardiac congestion or decongestion.	Study type/limitations are apparent from the screening record.
Erly et al., 2003	Not reported in screening record.	Not assessed/reported.	Not assessed/reported.	See abstract in screening record.	Additional evidence linking lymphatic structure/function with cardiac congestion or decongestion.	Study type/limitations are apparent from the screening record.
Slanetz et al., 1998	Not reported in screening record.	Not assessed/reported.	Not assessed/reported.	See abstract in screening record.	Additional evidence linking lymphatic structure/function with cardiac congestion or decongestion.	Study type/limitations are apparent from the screening record.

References

1. Wu et al. 2024 Lymphatic vessels are necessary for cardiac function and inflammation resolution in sepsis-induced cardiomyopathy. doi:10.1016/j.intimp.2024.113511.
2. Luo et al. 2026 Therapeutic Lymphangiogenesis Using Induced Cardiac Fibroblasts Protects the Heart From Heart Failure With Preserved Ejection Fraction Progression by Exerting Anti-Inflammatory and Antifibrotic Effects. 2026. doi:10.1253/circj.CJ-25-0757.
3. Vieira et al. 2018 The cardiac lymphatic system stimulates resolution of inflammation following myocardial infarction. doi:10.1172/JCI97192.

4. Ge et al. 2020 LCZ696, an Angiotensin Receptor-Neprilysin Inhibitor, Improves Cardiac Hypertrophy and Fibrosis and Cardiac Lymphatic Remodeling in Transverse Aortic Constriction Model Mice. doi:10.1155/2020/7256862.
5. Bai et al. 2022 Angiotensin II Induces Cardiac Edema and Hypertrophic Remodeling through Lymphatic-Dependent Mechanisms. doi:10.1155/2022/5044046.
6. Cui et al. 2023 Inhibition of PFKFB3 Expression Stimulates Macrophage-Mediated Lymphangiogenesis Post-Acute Myocardial Infarction. doi:10.31083/j.fbl2811277.
7. Xu et al. 2025 Network pharmacology and bioinformatics analysis reveals: NXC improves cardiac lymphangiogenesis through miR-126-3p/SPRED1 regulating the VEGF-C axis to ameliorate post-myocardial infarction heart failure. doi:10.1016/j.jep.2025.119959.
8. Houssari et al. 2020 Lymphatic and Immune Cell Cross-Talk Regulates Cardiac Recovery After Experimental Myocardial Infarction. doi:10.1161/ATVBAHA.120.314370.
9. Luo et al. 2026 Adipose-derived regenerative cell therapy mitigates sepsis-induced cardiomyopathy through the induction of cardiac reparative lymphangiogenesis. doi:10.1186/s13287-026-05023-3.
10. Chen et al. 2024 Macrophage-derived VEGF-C reduces cardiac inflammation and prevents heart dysfunction in CVB3-induced viral myocarditis via remodeling cardiac lymphatic vessels. doi:10.1016/j.intimp.2024.113377.
11. Su et al. 2025 VEGFC/VEGFR3 Signaling- Dependent Lymphatic Remodeling Modulates Cardiac Response to Pressure Overload. doi:10.1161/JAHA.124.040649.
12. Xiang et al. 2026 Dragon's blood and its active components ameliorate pressure overload-induced heart failure via remodeling of cardiac lymphatics. n.d.. doi:10.1016/j.jep.2026.121595.
13. Nakanishi et al. 2026 VEGF-C-mediated cardiac lymphangiogenesis promotes inflammation resolution in autoimmune acute myocarditis in mice. n.d.. doi:10.1093/cvr/cvag057.
14. Guo et al. 2025 Lymphatic Endothelial Branched-Chain Amino Acid Catabolic Defects Undermine Cardiac Lymphatic Integrity and Drive HFpEF. n.d.. doi:10.1161/CIRCULATIONAHA.124.071741.
15. Keller et al. 2021 Genetic blockade of lymphangiogenesis does not impair cardiac function after myocardial infarction. n.d.. doi:10.1172/JCI147070.
16. Lin et al. 2021 VEGF-C/VEGFR-3 axis protects against pressure-overload induced cardiac dysfunction through regulation of lymphangiogenesis. n.d.. doi:10.1002/ctm2.374.
17. Zhang et al. 2019 Enhancement of cardiac lymphangiogenesis by transplantation of CD34(+)VEGFR-3(+) endothelial progenitor cells and sustained release of VEGF-C. n.d.. doi:10.1007/s00395-019-0752-z.
18. Shimizu et al. 2018 Impact of Lymphangiogenesis on Cardiac Remodeling After Ischemia and Reperfusion Injury. n.d.. doi:10.1161/JAHA.118.009565.
19. Henri et al. 2016 Selective Stimulation of Cardiac Lymphangiogenesis Reduces Myocardial Edema and Fibrosis Leading to Improved Cardiac Function Following Myocardial Infarction. n.d.. doi:10.1161/CIRCULATIONAHA.115.020143.
20. Salah et al. 2025 Lymphatic Drainage in Patients with Heart Failure: A Feasibility Study. 2025. doi:10.1007/s12265-025-10592-4.
21. Liu et al. 2023 Strategies in activating lymphatic system on symptom distress and health-related quality of life in patients with heart failure: secondary analysis of a pilot randomized controlled trial. 2023. doi:10.3389/fcvm.2023.1248997.
22. Kelly et al. 2024 Lymphatic morphology and function in chronic right heart failure due to secondary tricuspid valve regurgitation. 2024. doi:10.1016/j.ijcard.2024.132399.
23. Gharzeddine et al. 2026 Randomized Controlled Trial Nurse-Led, Home Lymphatic Exercises on Fluid Overload with Heart Failure. n.d.. doi:10.1097/NNR.0000000000000932.
24. Dori et al. 2023 Ascites in Animals With Right Heart Failure: Correlation With Lymphatic Dysfunction. n.d.. doi:10.1161/JAHA.122.026984.
25. Chabbert et al. 2004 Mediastinal lymphadenopathy in congestive heart failure: a sequential CT evaluation with clinical and echocardiographic correlations. n.d.. doi:10.1007/s00330-003-2168-1.
26. Erly et al. 2003 Location, size, and distribution of mediastinal lymph node enlargement in chronic congestive heart failure. n.d.. doi:10.1097/00004728-200307000-00005.
27. Slanetz et al., 1998 Mediastinal lymphadenopathy and hazy mediastinal fat: new CT findings of congestive heart failure. n.d.. doi:10.2214/ajr.171.5.9798869.
Note: The screening file contains 38 records marked “Include”. The current primary Table 1 contains studies that are not all present in this screening file; therefore, the number of studies in this supplementary table (37) is not intended to be mathematically complementary to Table 1 until the screening file and primary table are reconciled. The entries above are derived from the supplied screening file and no additional eligibility decisions were introduced.
